# Cyanobacterial Harmful Algal Mats (CyanoHAMs) in tropical rivers of central Mexico and their potential risks through toxin production

**DOI:** 10.1007/s10661-024-12568-4

**Published:** 2024-04-02

**Authors:** Angela Caro-Borrero, Kenia Márquez-Santamaria, Javier Carmona-Jiménez, Itzel Becerra-Absalón, Elvira Perona

**Affiliations:** 1 Ecology and Natural Resources Department, Science Faculty, National Autonomous University of Mexico, University City, Exterior Circuit S/N, 04510 Coyoacan, Mexico City, Mexico; 2 Postgraduate School in Marine Sciences and Limnology, National Autonomous University of Mexico, University City, Exterior Circuit S/N, 04510 Coyoacan, Mexico City, Mexico; 3 Comparative Biology Department, Science Faculty, National Autonomous University of Mexico, University City, Exterior Circuit S/N, 04510 Coyoacan, Mexico City, Mexico; 4 Biology Department, Science Faculty, Autonomous University of Madrid, Darwin 2, Canto Blanco Campus, 28049 Madrid, Spain

**Keywords:** Benthic cyanobacteria; Cyanobacterial Harmful Algal Mats; Microcystins, Anatoxins, River growths, Polyphasic approach

## Abstract

Cyanobacteria inhabiting lotic environments have been poorly studied and characterized in Mexico, despite their potential risks from cyanotoxin production. This article aims to fill this knowledge gap by assessing the importance of benthic cyanobacteria as potential cyanotoxin producers in central Mexican rivers through: (i) the taxonomic identification of cyanobacteria found in these rivers, (ii) the environmental characterization of their habitats, and (iii) testing for the presence of toxin producing genes in the encountered taxa. Additionally, we introduce and discuss the use of the term “CyanoHAMs” for lotic water environments. Populations of cyanobacteria were collected from ten mountain rivers and identified using molecular techniques. Subsequently, these taxa were evaluated for genes producing anatoxins and microcystins via PCR. Through RDA analyses, the collected cyanobacteria were grouped into one of three categories based on their environmental preferences for the following: (1) waters with high ionic concentrations, (2) cold-temperate waters, or (3) waters with high nutrient enrichment. Populations from six locations were identified to genus level: *Ancylothrix* sp., *Cyanoplacoma* sp., and *Oxynema* sp. The latter was found to contain the gene that produces anatoxins and microcystins in siliceous rivers, while *Oxynema* tested positive for the gene that produces microcystins in calcareous rivers. Our results suggest that eutrophic environments are not necessarily required for toxin-producing cyanobacteria. Our records of *Compactonostoc*, *Oxynema*, and *Ancylothrix* represent the first for Mexico. Four taxa were identified to species level: *Wilmottia* aff. *murrayi*, *Nostoc tlalocii*, *Nostoc montejanii*, and *Dichothrix* aff. *willei*, with only the first testing positive using PCR for anatoxin and microcystin-producing genes in siliceous rivers. Due to the differences between benthic growths with respect to planktonic ones, we propose the adoption of the term Cyanobacterial Harmful Algal Mats (CyanoHAMs) as a more precise descriptor for future studies.

**Supplementary Information:**

The online version contains supplementary material available at 10.1007/s10661-024-12568-4.

## Introduction

Cyanobacteria are photosynthetic organisms found in almost every aquatic environment on the planet. One of the evolutionary strategies that allows them to compete for resources with other aquatic organisms is the synthesis of secondary metabolites called cyanotoxins, which can damage the metabolism and functioning of all kinds of living beings, including humans (Aguilera et al., 2023; Almuhtaram et al., 2021; Massey et al., 2022). The presence of some species of cyanobacteria, particularly when in large quantities (blooms), could imply an environmental, ecological, and human health risk (Rider et al., 2024; Schulte et al., 2022). In the scientific literature, the first mention of the term “bloom” dates back to 1953, when it was used to describe the massive growth of planktonic cyanobacteria in a lake (Sverdrup, 1953). The term is not exclusive to cyanobacteria, however, since it can refer to peaks of biomass or phytoplanktonic density (Frau, 2023). This leads to the term being widely used with different meanings, resulting in a definition that remains largely ambiguous (Aguilera et al., 2023). One of the relevant aspects in the massive growths of cyanobacteria is the production of secondary metabolites, of which more than 2000 have been characterized, including cyanotoxins (Jones et al., 2021). The presence of Cyanobacterial Harmful Algal Blooms (CyanoHABs) is generally related to problems of water contamination derived from anthropic activities that favor an increase in the concentration of nutrients such as phosphorus and nitrogen, as well as in relation to climate change, such as increases in water temperature and changes in the natural hydrological seasonal regimes due to longer periods of drought and periods with storm peaks (Agha et al., 2012; Buratti et al., 2017; Shahmohamadloo et al., 2020; Chorus et al., 2021; Reinl et al., 2021; Rider et al., 2024). As the main source of water for human activities comes from lentic environments, CyanoHABs have been a topic of great relevance (Almuhtaram et al., 2021; Massey et al., 2022; Quiblier et al., 2013). As a result, studies on cyanotoxins have been biased toward populations of planktonic cyanobacteria that inhabit lentic ecosystems (Aguilera et al., 2023; Almuhtaram et al., 2021). The cyanoHABs concept is almost always associated with populations of phytoplanktonic cyanobacteria in lakes and reservoirs, but riverbeds HABs being much less studied even though they have recently been recognized as important producers of toxins with effects on the food weber (Legleiter & Hodges, 2022; Rider et al., 2024).

In lotic environments, these massive growths develop at the bottom of the river in a variety of continental habitats and are often not visible due to the turbidity of the water or a high concentration of suspended solids. Furthermore, they are spatially and temporally variable and the timing and magnitude of the drivers of growth in benthic productivity have been described as varied and include streamflow velocity, light, nutrients, sediment deposition, substrate composition, and watershed characteristics (Rider et al., 2024; Schulte et al., 2022). Filamentous and mucilaginous colonies are usually the dominant growth forms with a higher report of anatoxins, microcystins, and saxitoxins production in rivers (Cantoral Uriza et al., 2017; Legleiter & Hodges, 2022; Loftin et al.^2016^). Reports of massive growths and their relationship with toxicity and bioaccumulation events at higher trophic levels such as benthic macroinvertebrates (Shahmohamadloo et al., 2020) and fish (Zamora-Barrios et al., 2019) are becoming more frequent, as well as the poisoning of domestic animals such as dogs and cattle (livestock) (Bouma-Gregson et al., 2019; Echenique-Subiabre et al., 2016; Gaget et al., 2020). In a review focusing on Latin America, Aguilera et al., (2023) found that 295 blooms were reported in the literature in freshwater ecosystems between 2000 and 2019. These reports include rivers from 14 countries, and 67% of the cases report an association with the presence of cyanotoxins. However, most of this information focused on lake ecosystems. Dissolved phosphorus and nitrogen in water have been recognized as limiting nutrients that, when in excess, can drive the growth of blooms and the biosynthesis of planktonic cyanobacterial toxins (Chorus et al., 2021; Reinl et al., 2021). Although this phenomenon has also been shown to be associated with oligotrophic water bodies, these systems have been less studied, causing a limitation in our understanding of the development and maintenance of blooms (Reinl et al., 2021; Rider et al., 2024; Schulte et al., 2022). In the case of benthic mats, the influence of nutrients is more complex due to the diverse biosynthetic capacities of the cyanobacterial species and associated bacteria in the biofilm (Bouma-Gregson et al., 2019; Robichon et al., 2023). For example, a metagenomic mapping of cyanobacteria mats in large rivers within the United States of America recorded Proteobacteria, followed by Actinobacteria, Bacteroidetes, and Cyanobacteria as the most abundant and frequent bacterial taxa in general (Linz et al., 2023). It is possible that the non-cyanobacterial organisms present in the environment influence the production and degradation of cyanotoxins (Bouma-Gregson et al., 2019).

The most widely reported cyanotoxins in cyanobacteria mats are microcystins and anatoxins (Almuhtaram et al., 2021; Rider et al., 2024). Microcystins are cyclic peptides that act as potent inhibitors of phosphatase enzymes and are frequently associated with liver damage and tumor growth (Chorus et al., 2021). Anatoxin-a is a neurotoxic alkaloid that can passively cross biological membranes and can act as a potent agonist of the nicotinic acetylcholine receptor, which inhibits neuromuscular receptors by disrupting cellular ion channels, resulting in muscle failure and sometimes death (Bouma-Gregson et al., 2018; Cadel-Six et al., 2007; Colas et al., 2021).

Most cyanotoxins are retained inside the cells, therefore their intake is required to trigger side effects. The type and quantity of toxins depend on the biological composition present in the bloom or mat, as well as the predominant toxigenic genotypes (Chorus et al., 2021). The latter refers to the presence of functional genes involved in the biosynthesis of particular toxins, which are usually found co-existing in situ with populations that are not toxigenic and are indistinguishable using conventional analyses such as light microscopy (Cadel-Six et al., 2007; Colas et al., 2021). For this reason, one of the main stages in the developing field of cyanotoxin research is the taxonomic identification of cyanobacterial populations. Identification under the polyphasic approach is a key tool in the establishment of new toxin-producing cyanobacteria species, not only because a single morphotype can be associated with more than one species but also because of the diversity of cyanotoxin congeners that differ between species. The detection of these blooms and the polyphasic approach is mainly focused on planktonic populations, where spatial and temporal changes in the appearance and maintenance of blooms constitute an important monitoring factor (Munoz et al., 2021).

Toxin production has been confirmed in at least twelve genera of benthic cyanobacteria (*Geitlerinema*, *Kamptonema*, *Lyngbya*, *Microcoleous*, *Microseira*, *Nostoc*, *Oscillatoria*, *Phormidium*, *Rivularia*, *Schizothrix*, *Tolypothrix*, and *Tychonema*) by ELISA and/or HPLC–PDA (Aboal & Puig, 2009; Aboal et al., 2005; Bauer et al., 2022; Conklin et al., 2022; Seifert et al., 2007; Valdor & Aboal, 2007; Wood et al., 2018, 2020), HPLC–PDA and HPLC–MS (Cantoral Uriza et al., 2017), and less frequently by genetic methods (gene amplification of mcyE and anaF through PCR) (Amador, 2019). Detection of these genes is currently being developed and applied in lake risk assessment monitoring programs (Dittmann & Börner, 2005; Rantala-Ylinen et al., 2011).

In Mexico, studies on cyanotoxins in lentic environments are slowly emerging, but those examining lotic environments are lacking (Backer, 2002). Studies involving the phenotypic, genotypic, and ecological characterization of potentially cyanotoxin-producing populations are an important tool for protecting the ecological integrity of water bodies and the potential negative effects on animals and humans (e.g., rural/indigenous communities inhabiting riverbanks). As such, this article aims to assess the importance of benthic cyanobacteria as potential producers of cyanotoxins in lotic environments within the tropical regions of central Mexico through: (i) taxonomic identity (phenetic and genetic) of recurrent cyanobacteria mats in the region; (ii) environmental characterization of sites where they are found; and (iii) the presence of cyanotoxin-expressing genes. Additionally, we introduce and discuss the use of the term “CyanoHAMs” to refer to benthic mats potentially related to cyanotoxin production in lotic water bodies, differentiated from planktonic blooms (“CyanoHABs”).

## Materials and methods

### Study area

**Fig. 1:**
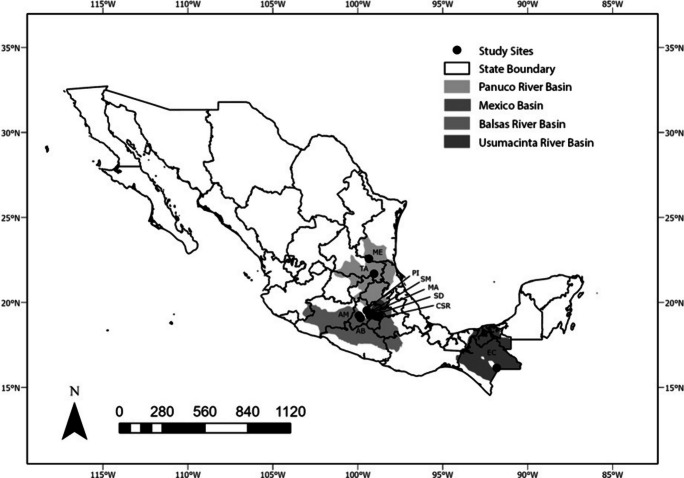
Study sites with cyanobacterial mats in central and southeastern Mexico. Monte Alegre (MA), Segundo Dinamo of Magdalena river (SD), Iturbide Dam (PI), San Miguel (SM), San Rafael Channel (CSR), Arroyo Monarca (AM), Agua Blanca (AB), Meco (ME), Tambaque (TA), and Carrizal (EC)

Central and southeastern Mexico (16–23° LN, 91–100° LW) is a heterogeneous region that includes mountain rivers (above 2500 m) of volcanic origin in temperate forests (Cuevas et al., 2010; García, 2004), as well as lowland streams of calcareous origin in tropical rainforests (Cuevas et al., 2010; Soares & García, 2017). Fieldwork was conducted from May 2018 to June 2022 to collect samples, including from ten of the most resilient cyanobacterial populations (Fig. 1) representing the major basins in the country (Bojorge García et al., 2010, Rodriguez-Flores and Carmona-Jiménez, 2018, Cartajena-Alcántara et al., 2020).

### Environmental characterization

Temperature, pH, dissolved oxygen levels, and specific conductance were measured with a YSI 6600 multiparameter (Ohio, USA). To assess water chemistry, one-liter samples were taken in duplicated and nutrient concentrations were analyzed in the field within one hour of collection using a DRELL 3000 laboratory (Hach Company, Colorado, USA). Soluble reactive phosphorous (SRP), nitrite nitrogen (N- NO_2_), nitrate nitrogen (N-NO_3_), and ammonium nitrogen (N-NH_4_) were analyzed following the Standard Methods for the Examination of Water and Wastewater (American Public Health Association (APHA), 1995). Dissolved inorganic nitrogen (DIN) was calculated as the sum of the three inorganic nitrogen forms present in the samples.

### Statistical analysis

A Redundancy Analysis (RDA) was performed to assess the relationship between the study populations and the environmental variables of each site. Such relationships could indicate specific ecological preferences of each cyanobacteria and illuminate variables linked to optimal growth, proliferation, and potential toxin production of cyanobacterial mats in rivers. The analysis was performed using the statistical package PAST 3.x (Hammer, 2013) with a data transformation of log10(*x*) + 1.

### Sampling collection

At each sampled rivers, visible growths of cyanobacterial mats were collected in triplicate for subsequent morphological and molecular analyses in the laboratory. The collected material was reviewed under an optical microscope to identify morphological structures useful for species identification following specialized bibliographic resources (Broady & Ingerfeld, 1991; Komárek & Anagnostidis, 2005; Komárek, 2013; Strunecký et al., 2011; Martins et al., 2016). For taxonomic analyses, an Olympus BX51 microscope with a DP12 digital camera (Olympus, Tokyo, Japan) was used.

### DNA extraction and sequencing

DNA was extracted from samples using a QIAGEN DNeasy UltraClean Microbial Extraction Kit. Frozen mat samples were thawed at room temperature for 0.5 h, and ~ 0.15 g of mat was removed for DNA extraction. DNA extraction followed the manufacturer’s protocol, except for a modified cell lysis step. The samples were pretreated to facilitate cell rupture, which consisted of three freeze/heat cycles with liquid nitrogen and heating in an AccuBlock (Labnet International Inc.) at 65 °C. Between each cycle, a drill and a plastic pistil were used to mechanically break up the mat. The presence of DNA was confirmed by electrophoresis (0.8% agarose gel), and DNA concentrations were measured in a microplate spectrophotometer (Epoch: BioTek Instruments Inc., USA).

### PCR amplification of the 16S rRNA gene

Amplification of the 16S rRNA gene was performed by PCR using the Biometra TOne Thermal Cyclers thermocycler (Analytik Jena; Göttingen, Germany). The following reaction master mix was used: milli-Q water, 10 × PCR buffer, Cl_2_Mg (50 mM), deoxyribonucleotide triphosphate (50 μM dNTP), bovine serum albumin (BSA, 0.1%), DNA polymerase (Ultratools DNA Polymerase: 1 unit/μL, and Thermoscientific DreamTaq DNA Polymerase: 20 and 500 unit/μl). The following primers were used at 10 pM: 27F (5″—AGAGTTTGATCCTGGCTCAG-3′) (Wilmotte et al., 1994) and 23Sr (5′- CTTCGCCTCTGTGTGCCTAGGT-3′) (Lepère et al., 2000). Milli-Q water was used as negative control. Agarose gel electrophoresis (1.5%) was performed to reveal visualize PCR products. Once DNA bands were confirmed, the PCR product was purified using the Wizard SV Gel and PCR Clean-up System Kit (Promega). Subsequently, a cloning procedure was performed using the Pgem-t Easy Vector System Ligation Kit (Promega) to ensure the greatest biological representativeness of the algal consortium. The transformation process was carried out using 100 µL of the competent bacteria strain *Escherichia coli* (DH5a). The transformed bacteria were inoculated (250 μL) in Petri dishes with solid LB medium (Bertani et al*.*, 1951), ampicillin (0.1 mg/mL), X-Gal: 5-bromo-4-chloro-3-indolyl-β-d-galactopyranoside (0.04 mg/mL) and IPTG: isopropyl-β-d-1-thiogalactopyranoside (0.5 mM). The cultures were incubated at 37 °C for 24 h. Positive clones (presence of the insert) were recognized by their white coloration and subsequently reseeded in LB medium. Negative clones (blue colonies without insert) were discarded. The presence of the insert was confirmed by PCR and electrophoresis. The reaction master mix was comprised of milli-Q water, 10 × PCR buffer, Cl_2_Mg (50 mM), deoxyribonucleotide triphosphate (50 μM dNTP), and DNA polymerase (Ultratools DNA Polymerase: 1 unit/μL and Thermoscientific DreamTaq DNA Polymerase: 20 and 500 unit/μl). The primers T7 (5′-TAATACGACTCACTATAGGG-3′) and SP6 (5′-ATTTAGGTGACACTATAG-3′) (Melton et al., 1984) were used at 10 pM.

### Obtaining sequences

Once the presence of the insert was confirmed, the transformed bacteria were cultured in liquid LB medium with ampicillin at 37 °C and horizontal movement of 250 rpm for 24 h. Afterwards, the plasmids were extracted and purified using the Wizard Plus SV Minipreps DNA Purification System Kit (Promega). Once the plasmid DNA was obtained, final concentrations were measured (Epoch: BioTek Instruments Inc., USA) before sending for sequencing. Sequencing was carried out at the DNA Synthesis and Sequencing Unit (USSDNA) at the Institute of Biotechnology-UNAM (National Autonomous University of Mexico) and the Complutense University of Madrid (Genomic Unit-CAI). The sequencing primers were T7 (5′-TAATACGACTCACTATAGGG-3′), SP6 (5′-ATTTAGGTGACACTATAG-3′) (Melton et al., 1984), and 684F (5′-GTGTAGCGGTGAAATGCGTAGA-3′) (Nübel et al., 1997).

### Toxin detection

To evaluate the potential toxicity of cyanobacteria samples, PCR tests were carried out to amplify and assess genes involved in the production of cyanotoxins, specifically the *ana*F and *mcy*E genes. Amplification of toxin-producing genes was carried out following the same protocol used for the 16S rRNA gene. The reaction mixture used for the PCRs included: milli-Q water, 10 × PCR buffer, Cl_2_Mg (50 mM), deoxyribonucleotide triphosphate (dNTP 50 μM), (BSA, 0.1%), and DNA polymerase (Ultratools DNA Polymerase: 1 unit/μL and Thermoscientific DreamTaq DNA Polymerase: 20 and 500 unit/μL). Primers used to amplify anatoxins (10 pM) were atxof (5′-TCGGAAGCGCGATCGCAAATCG-3′) and atxar (5′- GCTTCCTGAGAAGGTCCGCTAG.3′) (Ballot et al., 2010), while HEPF (5′- TTTGGGGTTAACTTTTTTGGGCATAGTC-3′) and HEPR (5′- AATTCTTGAGGCTGTAAATCGGGTTT-3′) were used to amplify microcystins (Jungblut et al., 2006). Milli-Q water was included as a negative control.

### Phylogenetic analysis

The obtained sequences were assembled using the BioEdit 7.2 program (Hall, 2011) to produce consensus sequences for a subsequent BLAST analysis (Basic Local Alignment Search Too): https://blast.ncbi.nlm.nih.gov/Blast.cgi. Sequences with identity percentages above 98% similarity were considered matches. Together with the results of the morphological identification, a taxonomic identity was obtained for the populations of cyanobacteria analyzed. A phylogenetic tree was estimated with the sequences obtained from each study populations and the similar sequences from the BLAST analysis, as well as additionally sequences of some genera belonging to nearby taxonomic groups (http://www.cyanodb.cz/ and https://www.ncbi.nlm.nih.gov/). Once the sequence matrix had been constructed, a multiple alignment was performed using the BioEdit program, which was subsequently revised manually using the PhyDE-1 V 0.9971 program (Müller et al., 2010) under a maximum parsimony criterion. The phylogenetic estimation was carried out in the MEGA V.11.0.13 program (Tamura et al., 2021), using the maximum likelihood algorithm.

## Results

### Environmental characterization

**Fig. 2:**
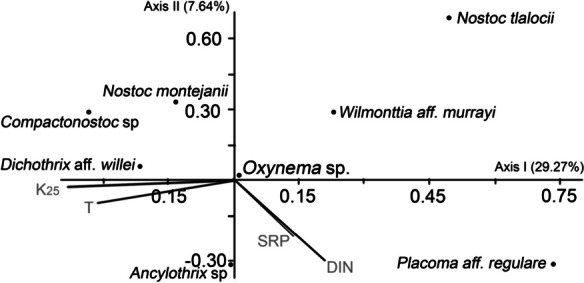
Redundancy Analysis (RDA) of the cyanobacterial mats in rivers from central Mexico. Sites and abbreviations of chemical and physical parameters are listed in Table 1

According to the RDA (Fig. 2), three groups can be recognized within the study populations based on environmental preferences. The first two axes explain 36.91% of the total variance. Axis I (29.27%) is correlated to specific conductance (K_25_) and temperature (T), grouping cyanobacteria that prefer waters with a higher concentration of ions (specific conductance 141–1528 µS/cm) such as *Dichothrix* aff*. willei, Nostoc montejanii*, and *Compactonostoc* sp., as well as those that prefer cold-temperate waters (7.6–15.1 °C) such as *Nostoc tlalocii*, *Wilmottia* aff. *murrayi*, and *Cyanoplacoma* sp. Axis II (7.64%) is correlated with soluble reactive phosphorus (SRP) and dissolved inorganic nitrogen (NID), indicating a group of cyanobacteria that prefer waters with greater enrichment of these nutrients (SRP 0.07–0.95 mg/L, NID 0.263–33.17 mg/L), including *Ancylothrix* sp. and *Oxynema* sp. (Table 1, Fig. 2).

**Table 1:** Physical and chemical parameters evaluated in rivers with cyanobacterial mats from central and southeastern basins of Mexico. *T*, temperature; *TDS*, total dissolved solids; *K*_*25*_, specific conductance; *DO*, dissolved oxygen; *SRP*, soluble reactive phosphorus; *NID*, dissolved inorganic nitrogen, *N*-*NH*_*4*_, ammonium; *N-NO*_*2*_, nitrites; *N-NO*_*3*_, nitrates. Land use: *HS*, human settlement; *TF*, tropical forest; *RA*, rainfed agriculture; *MF*, mountain mesophilic. Forest; *D*, dams

Basin and site	CyanoHAMs Taxonomic identity (16S gene amplification)	Percent coverage (%)	T	pH	K_25_ (µS/cm)	DO (mg/L)	SRP	N-NH_4_	N-NO_2_	N-NO_3_	NID	NID:SRP ratio	Land use
*Mexico Basin*													
1. Monte Alegre (MA)	*Nostoc tlalocii*	70	7.6	8.0	52	8.9	0.275	0.095	0.010	0.087	0.192	0.7	MF
2. Magdalena River (SD)	*Cyanoplacoma* sp. *Wilmottia* aff*. murrayi*	90 20	9.2	6.5	50	8	0.33	0.015	0.005	1.25	1.27	3.8	MF/HS
3. Iturbide Dam (PI)	*Nostoc tlalocii*	30	9.5	7.5	58	7.9	0.445	0.01	0.025	0.001	0.036	0.08	MF
4. San Miguel (SM)	*Compactonostoc* sp. *Cyanoplacoma* sp. *Wilmottia* aff*. murrayi*	40 15 30	14.3	6.2	74	8	0.74	0.15	0.003	0.025	0.178	0.2	MF
5. San Rafael Channel (CSR)	*Compactonostoc* sp.	30	9.9	7.2	141	7.9	0.375	0.03	0.003	0.01	0.043	0.1	MF/D
*Balsas Basin*													
6. Arroyo Monarca (AM)	*Cyanoplacoma.* sp.	15	12.7	6.2	90	8	1.05	0.12	0.006	1.6	1.706	1.6	MF/D
7. Agua Blanca (AB)	*Cyanoplacoma.* sp	40	15.1	7.1	176	9	1.41	0.33	0.005	1.7	0.585	0.4	MF/HS
*Panuco Basin*													
8. Meco (ME)	*Oxynema* sp. *Dichothrix* aff. *willei*	2 35	25.6	7.3	1215	6.1	0.07	0.01	0.003	0.25	0.263	3.7	HS
9. Tambaque (TA)	*Nostoc montejanii*	30	23.5	6.9	1528	8.4	0.02	0.06	0.002	0.035	0.097	4.8	TF
*Usumacinta Basin*													
10. Carrizal (EC)	*Ancylothrix* sp.	20	23.7	7.8	602	7.7	0.955	3.18	0.46	29.99	33.17	35.2	RA

### Taxonomic characterization

**Fig. 3:**
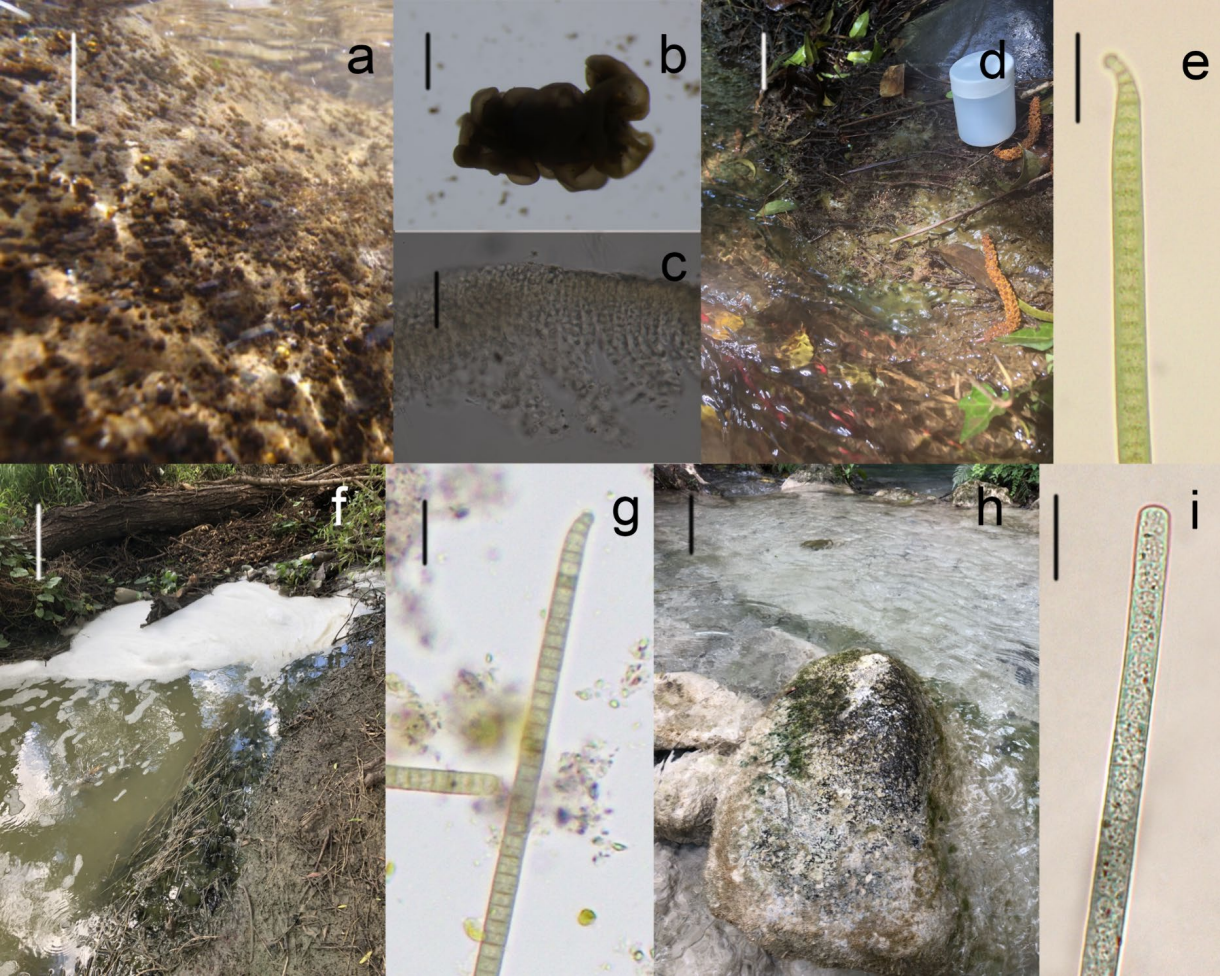
Freshwater cyanobacteria mats in Mexico. **a** Macroscopic colonies of *Cyanoplacoma* sp., **b** their mucilaginous colony, and **c** transversal view of colony. **d** Habitat of *Wilmottia* aff. *murrayi* and **e** apical filament with calyptra. **f** Habitat of *Ancylothrix* sp. and **g** apical filament with calyptra. **h** Habitat of *Oxynema* sp. and **i** apical filament with calyptra. Scale bar: Figs. **c**, **d**, **f**, and **h** = 10 cm; Fig. **d** = 1 cm; Figs.** c**, **e**, **g**, and **i** = 10 μm

**Fig. 4:**
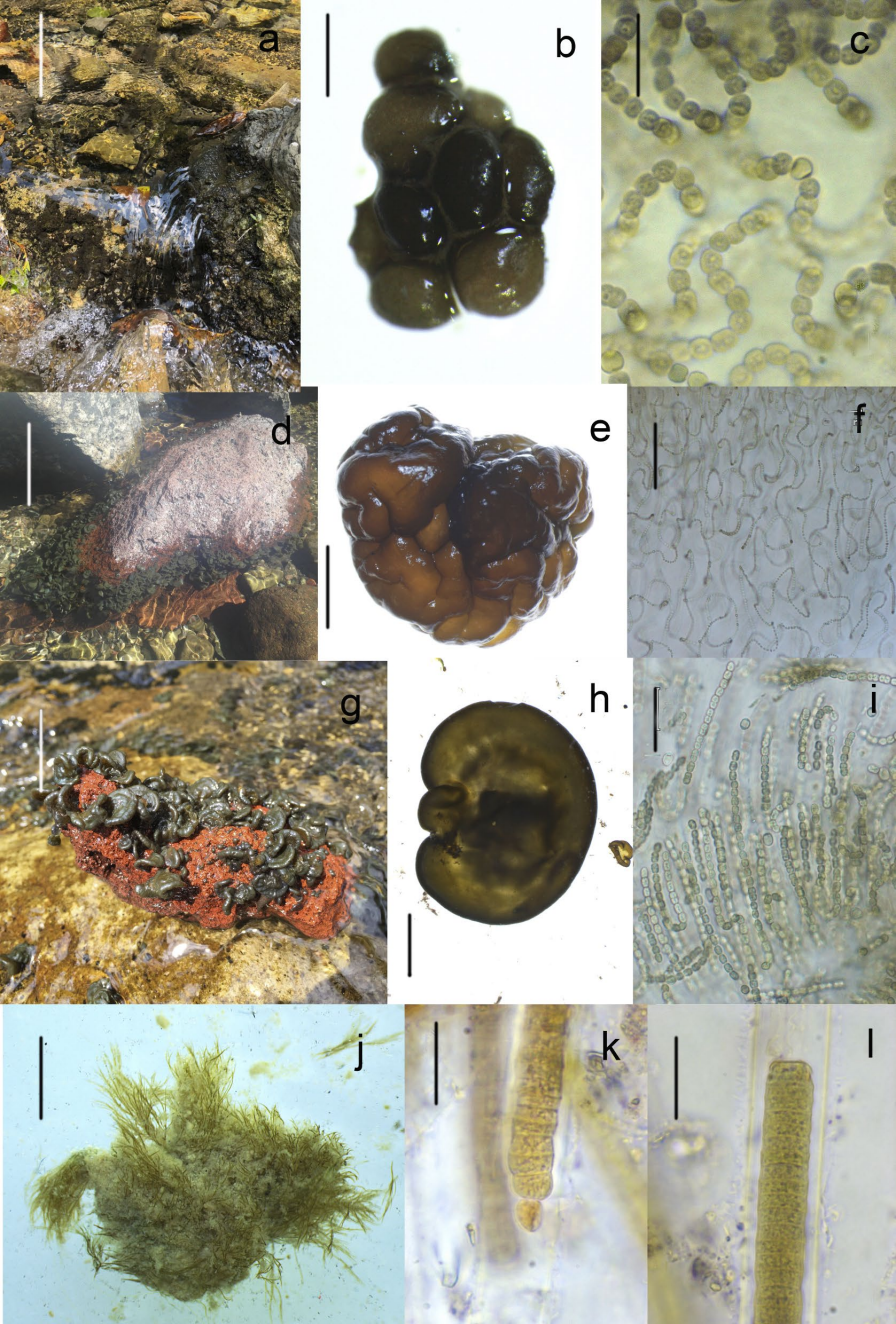
Freshwater cyanobacteria mats in Mexico. **a** Macroscopic colonies of *Nostoc montejanii*, **b** mucilaginous colony, and **c** transversal view of colony. **d** Habitat of *Nostoc tlalocii*, **e** mucilaginous colony, and **f** transversal view of colony. **g** Habitat of *Compactonostoc* sp., **h** mucilaginous colony, and **i** transversal view of colony. **j** Mats of *Dichothrix* aff. *Willey*, **k** false branch and heterocyte, and **l** apical filament and yellow sheath. Scale bar: **a**, **b, g**, and **k** = 10 cm; **b**, **e**, and **h** = 1 cm; **c**, **f**, **i**, and **l** = 10 μm

The taxonomic characterization of the cyanobacteria assessed in this work included different levels of analyses and types of information. This demonstrates the importance of employing a polyphasic approach for determining taxonomic identities, since a greater number of characters analyzed results in a greater level of taxonomic resolution. Amplification of the 16S gene in combination with morphological characterization resulted in eight different taxa (Figs. 3, 4, Supplementary material), distributed across three orders (Chroococcales, Oscillatoriales, and Nostocales), five families (Entophysalidaceae, Coleofasciculaceae, Microcoleaceae, Nostocaceae, and Rivulariaceae), and seven genera (*Cyanoplacoma*, *Wilmottia*, *Ancylothrix*, *Oxynema*, *Nostoc*, *Compactonostoc*, and *Dichothrix*). Taxonomic identification was established to the genus level in almost all populations using morphological and phylogenetic analyses. The morphological characteristics allowed all populations to be identified to the genus level (Table 2) with clear diagnostic characters, but at the specific level the morphological characters were only able to identify *Nostoc tlalocii*, *Nostoc montejanii*, *Wilmottia* aff*. murrayi*, and *Dichothrix* aff. *willei*. The other four characterized species very likely represent new species that will require more detailed taxonomic studies.

**Table 2:** Taxonomic identity of the dominant cyanobacteria found in field mats obtained through the molecular analysis and Basic Local Alignment Search tool. Presence ( +) or absence (-) of band in gel electrophoresis indicative of microcystin (*mcy*E) and/or anatoxin (*ana*F) genes evaluated through PCR analysis

Basin and site	CyanoHAMs Taxonomic identity (16S gene amplification)	*mcy*E gene	*ana*F gene
*Mexico Basin*			
1. Monte Alegre	*Nostoc tlalocii*	-	-
2. Magdalena River	*Cyanoplacoma* sp. *Wilmottia* aff*. murrayi*	- -	- +
3. Iturbide Dam	*N. tlalocii*	-	-
4. San Miguel	*Compactonostoc* sp. *Cyanoplacoma* sp. *Wilmottia* aff*. murrayi*	- + -	- + -
5. San Rafael channel	*Compactonostoc* sp.	-	-
*Balsas Basin*			
6. Arroyo Monarca	*Cyanoplacoma* sp.	-	+
7. Agua Blanca	*Cyanoplacoma* sp.	-	+
*Panuco Basin*			
8. Meco	*Oxynema* sp. *Dichothrix* aff. *willei*	- -	+ -
9. Tambaque	*Nostoc montejanii*	-	-
*Usumacinta Basin*			
10. Carrizal	*Ancylothrix* sp.	-	-

**Fig. 5:**
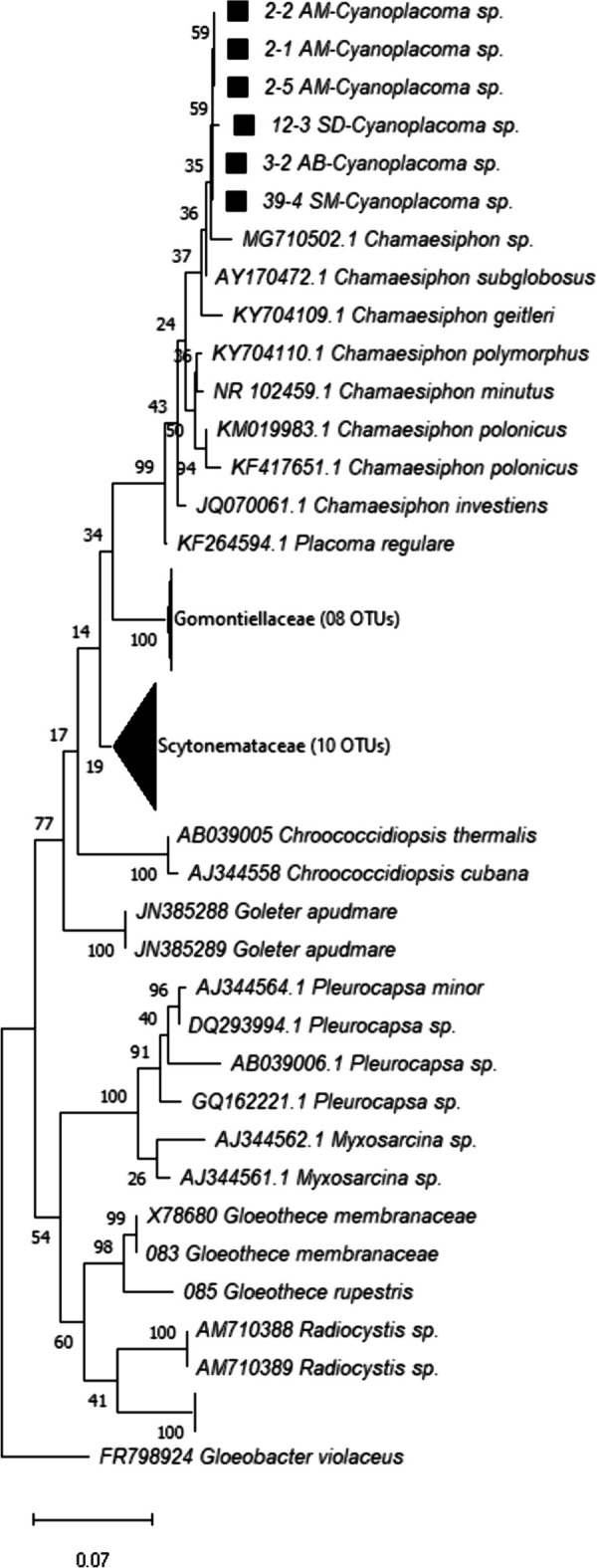
Phylogeny of the cyanobacteria genus *Cyanoplacoma*. Sequences marked with a black square correspond to the sequences obtained by amplification of the 16S gene in this study

The molecular phylogenetic reconstructions distinguished different populations that might be interpreted as the same genus with the morphology data. Specifically, the phylogenetic analysis indicated that populations from the sites SM, AM, and AB form a clade that belong to the *Cyanoplacoma* genus (BV: bootstrap value = 75). Additionally, short branch lengths separating the newly generated sequences suggest that they belong the same species (BV = 100) (Fig. 5), morphologically diagnosable as *Placoma regulare*, a genus that was recently renamed (Broady & Ingerfeld, 1991; Molinari-Novoa et al., 2021) as *Cyanoplacoma*. However, our sequences do not form a sister clade with *Placoma regulare*, suggesting that further taxonomic analyses are necessary to clarify their taxonomic identity.

**Fig. 6:**
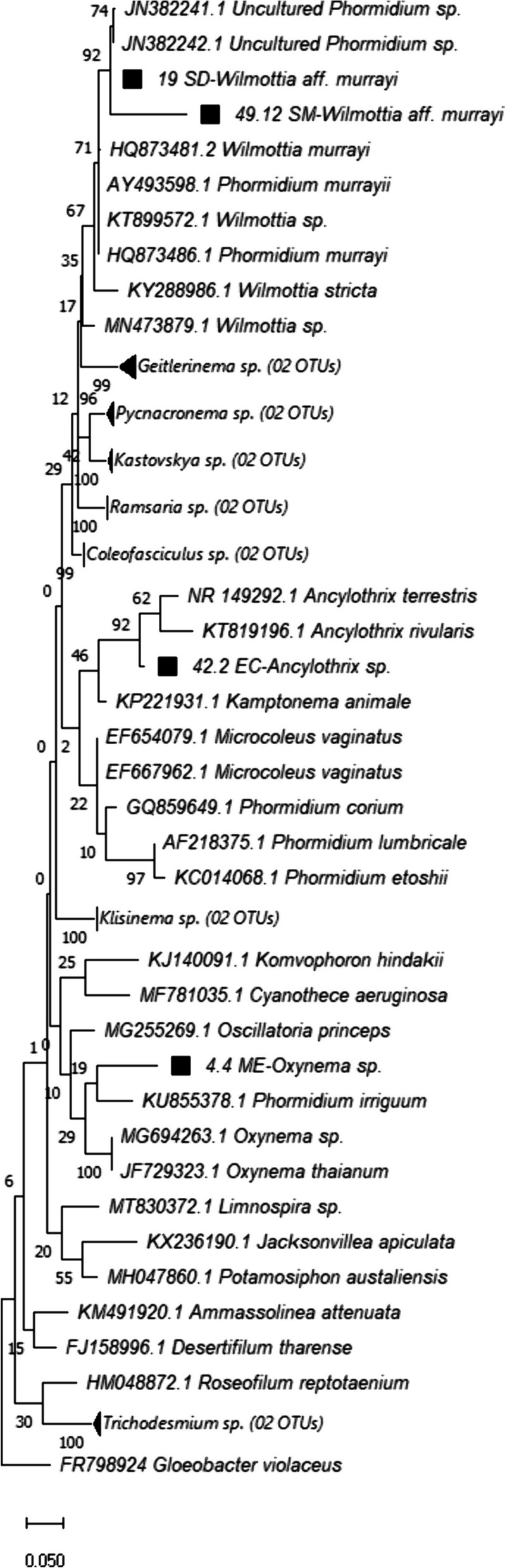
Phylogeny of the cyanobacteria order Oscillatoriales. Sequences marked with a black square correspond to the sequences obtained by amplification of the 16S gene in this study

The phylogeny of the Oscillatoriales group revealed that the populations from the SD and SM sites pertain to the genus *Wilmottia* (BV = 79), with a diagnosis consistent with *Wilmottia murrayi*. The ME population was recovered as sister to the genus *Oxynema* (BV = 51), which are similar in their filament termination and thylakoids, but this genus has only been reported from halophytic environments (Chatchawan et al., 2012). Considering this in combination with the low bootstrap value (Fig. 6), additional taxonomic analyses are necessary to clarify the taxonomic identity of the ME population. Finally, the population from the EC site is recovered as closely related to the genus *Ancylothrix* (BV = 97). This genus has only two described species, one of which is reported from benthic environments in rivers, leaving the possibility that the EC population is this same species or possibly a new species (Martins et al., 2016).

**Fig. 7:**
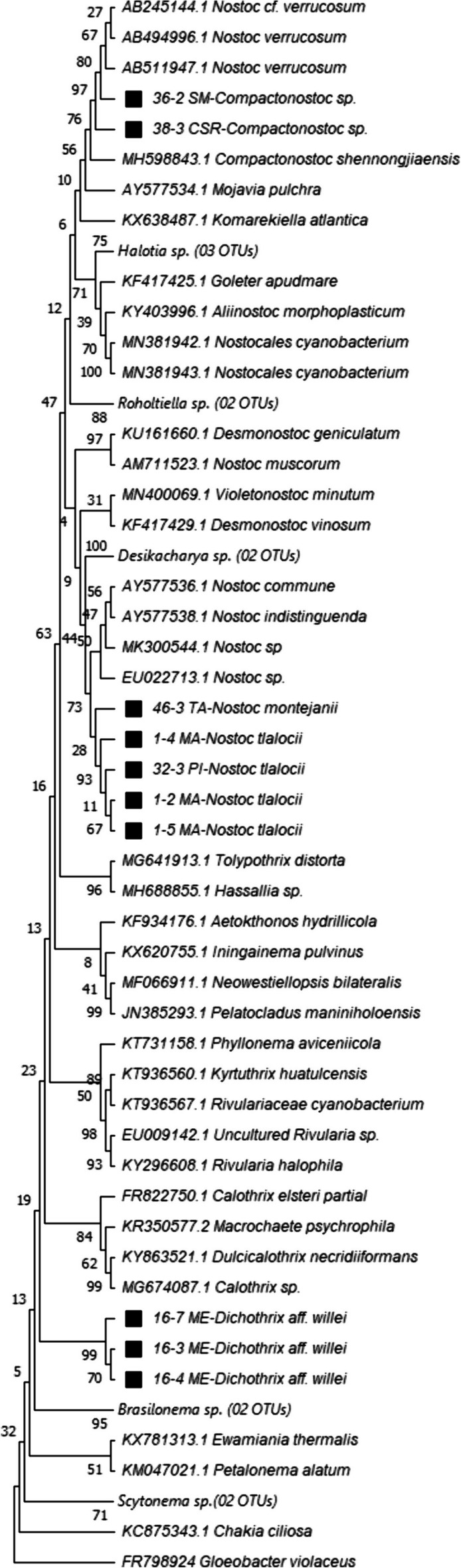
Phylogeny of the cyanobacteria order Nostocales. Sequences marked with a black square correspond to the sequences obtained by amplification of the 16S gene in this study

The phylogeny of the Nostocales group confirms that two of the populations originally associated by morphology with *Nostoc* do indeed belong to this genus (BV = 99%). As for their species identity, the morphological and phylogenetic data indicate that both the Monte Alegre and Iturbide populations belong to *N*. *tlalocii* (BV = 100%), while the Tambaque population (BV = 53) represents *N*. *montejanii* (Carmona-Jiménez et al., 2023). Interestingly, populations from San Rafael and San Miguel showed morphological characteristics similar to *Nostoc*, but the phylogenetic analysis indicated that these belong to the *Compactonostoc* genus. These populations present diagnostic characteristics of *Compactonostoc* in the form of macroscopic colonies that are amorphous, fine, and thin in shape, mucilaginous in consistency, and an olive green color, with trichomes uniseriate or biseriate only in short segments. *Compactonostoc* only has one species described, *C*. *shennongjiaensis*. Although measurements of the cells and heterocytes of this species overlap with our populations, the cells of *C*. *shennongjiaensis* tend to be smaller, so more taxonomic analyses are necessary to clarify the taxonomic identity of these individuals, they constitute a highly supported clade with a few other sequences labeled Nostoc (BV = 99%) that forms a sister clade to the Compactonostoc sequence (BV = 95%), but they probably represent a new species. Finally, samples from the ME site are recognized as members of the Rivulariaceae family, proposed here as *Dichothrix* aff. *willei* based on their morphological resemblance to this species (Fig. 7).

### Toxin detection

**Fig. 8:**
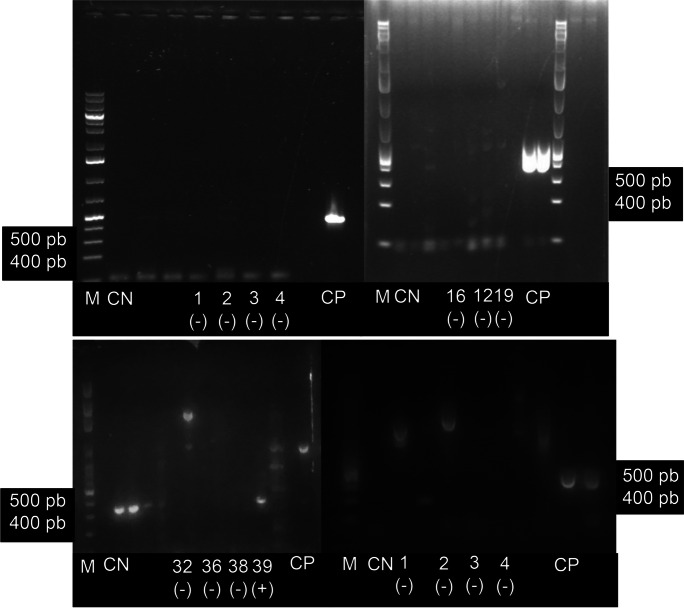
Electrophoresis gels showing PCR products for the identification of microcystins. (1) MA-*Nostoc tlalocii* (2) AM-*Cyanoplacoma* sp. (3) AB-*C.* sp. (4) ME- *Oxynema* sp. (12) SD- *C.* sp. (16) ME-*Dichothrix* aff *willei*. (19) SD-*Wilmottia* aff. *murrayi*. (32) PI-*Nostoc* tlalocii (36) SM*-Compactonostoc* sp*.* (38) CSR-*Compactonostoc* sp*.* (39) SM- *C.* sp. (42) EC-*Ancylothrix* sp. (46) TA-*Nostoc montejanii* (49) SM-*Wilmottia* sp. M: molecular marker. CN: negative control. CP: positive control

**Fig. 9:**
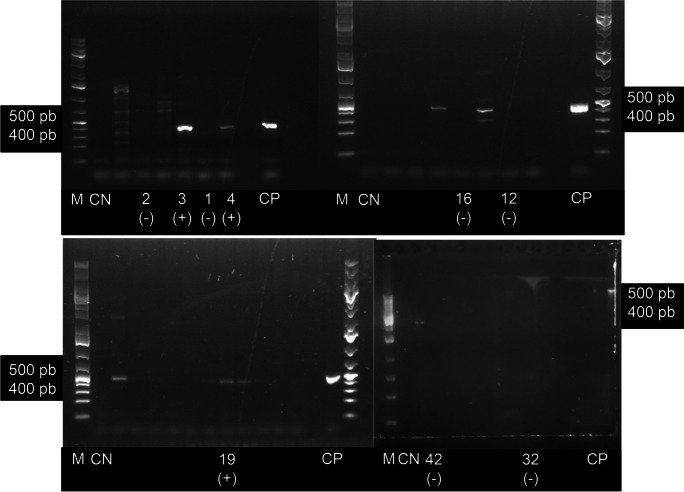
Electrophoresis gels showing PCR products for the identification of anatoxins. (1) MA-*Nostoc tlalocii* (2) AM-*Cyanoplacoma* sp. (3) AB-*C.* sp. (4) ME-*Oxynema* sp. (16) ME-*Dichothrix* aff. *willei*. (19) SD-*Wilmottia* aff. *murrayi*. (32) PI-*Nostoc tlalocii* (42) EC-*Ancylothrix* sp. M: molecular marker. CN: negative control. CP: positive control

The PCR tests found at least three samples from siliceous rivers to possess the ability to produce cyanotoxins (Table 3; Figs. 8 and 9). In the *Cyanoplacoma* sp. sample from San Miguel (SM), the *mcy*E (a microcystins biosynthetic gene) was amplified and detected in the band close to 472 bp. Meanwhile, in both samples of *Cyanoplacoma* sp. from San Miguel and from Agua Blanca (AB), as well as in the *Wilmottia* aff. *murrayi* sample from Segundo Dinamo (SD), the *ana*F, a anatoxin biosynthetic gene, was amplified and detected in the band close to 461 bp. As for samples from the calcareous rivers, the *ana*F gene was only amplified and detected in *Oxynema* sp. from Meco (ME).

Proposal of the term “cyanoHAMs:” According to our results, the growths of benthic cyanobacteria in rivers do not present patterns similar to those defined for planktonic blooms. Therefore, the identification of a potentially dangerous growth due to the production of toxins presents different characteristics typical of riparian environments. Here, we define cyanoHAMs (Cyanobacterial Harmful Algal Mats) as visible growths on river bottoms that can occupy large areas of the riverbed. However, given that morphological cyanobacterial forms include mats (flat composed of tightly interwoven filaments), gelatinous colonies (flat or semierect thallus with numerous cells or filaments in a common matrix), free filaments (semierect individual filaments without a matrix), and crusts (flat thallus composed of compacted tiers of cells) of benthic species (Sheat & Cole, 1992), the newly proposed term can also refer to abundant growth on different substrates within the river channel. CyanoHAMs do not necessarily cover the entire river benthos, and they can be found on the shore or in the center of the channel depending on the ecology of the particular species. A fundamental difference in contrast to CyanoHABs, given the nature of rivers and their constantly moving water is that cyanoHAMs do not normally change the color (blue-green) of the water associated with growth. In this sense, we agree with the proposal of Quiblier et al., (2013) that suggests evaluating the risk of massive benthic growths based on percent coverage. In this investigation, the cyanobacterial percent coverage ranged from 15 to 90% (with one outlier of 2% coverage). Another important characteristic of cyanoHAMs, in addition to the percent coverage, is whether the mats are attached to the substrate or have become detached so that the biomass accumulates on the banks or backwater areas. Detachment evidences that benthic growth is massive.

## Discussion

The massive growths of benthic cyanobacteria in tropical regions of Mexico were related to a combination of physical and chemical parameters that were different in space and time for each species. First, the geological origin of each basin serves as the principal defining element for each species’ preference for either carbonated or silicic waters, while water concentrations of DIN and SRP are secondary elements. All benthic cyanobacteria grow on firm substrates that can vary in size, always submerged and in well-oxygenated waters with a continuous flow, making them resilient inhabitants of the rivers they inhabit (Rodriguez-Flores & Carmona-Jiménez, 2018, Cartajena-Alcántara et al., 2020). In calcareous regions, *Nostoc montejanii* and *Oxynema* sp. can grow in oligotrophic waters, while *Ancylothrix* sp. can grow in eutrophic waters. Meanwhile, in the siliceous basins *Nostoc tlalocii*, *Compactonostoc* sp., and *Cyanoplacoma* sp. were recorded in oligotrophic waters, while *Wilmottia* aff. *murrayi* was found in mesotrophic waters. The wide heterogeneity in which massive growths can occur is related to the various morphological and functional adaptations for surviving in extreme conditions, such as the absence of nutrients (e.g., *Nostoc*, *Cyanoplacoma*, and *Dichothrix*), as well, a high concentration of DIN and SPR dissolved in the water (for example, *Ancylothrix* and *Wilmottia*). For example, phosphorus limitations are an important environmental and biological factor that, under limiting conditions, can trigger competition processes with other fixing bacteria (Reinl et al., 2021). Similar results have been seen in other studies, which even show that benthic cyanobacteria can grow by adapting to specific river conditions, influenced in principle by hydrological regimes (Robichon et al., 2023). Demonstrating that beyond responding to poor concentrations of nutrients, they can respond to larger scale and longer-term changes (Legleiter & Hodges, 2022). In this sense, our results contribute to the understanding of the ecology of proliferations in oligotrophic environments and the secondary metabolism related to toxin production (Aguilera et al., 2023; Reinl et al., 2021).

These particular responses are related to the autoecology of the species; therefore, a relevant aspect was the taxonomic validation through phylogenetic reconstructions in order to clarify and the taxonomic identity and rectify the findings obtained through the BLAST analyses. Since the latter are exploratory approaches that often present problems including erroneous identifications or very short sequences in the database that yield unreliable consensus results. Both the morphological and molecular data are consistent with the samples from Monte Alegre and Iturbide belonging to the genus *Nostoc*, specifically to *N*. *tlalocii*, while the Tambaque population is identified as *N*. *montejanii*. Both species were recently described by Carmona-Jiménez et al., (2023).

Meanwhile, the populations from CSR and SM pertain to *Compactonostoc* based on both morphological and phylogenetic evidence. Comparing these samples with the diagnostic characteristics of *C*. *shennongjiaensis* (Cai et al. 2019), our populations likely represent a new species, but further taxonomic analyses are necessary to validate this. Notwithstanding, this finding is important because it represents the first record of *Compactonostoc* from Mexico. *Nostoc tlalocii* and *Compactonostoc* sp. have an affinity for cold-water silicic rivers, while *N*. *montejanii* prefers warm-water calcareous rivers.

The *Cyanoplacoma* genus was confirmed in our study populations by their characteristic morphology in the shape of the thallus and the cell division pattern in the conformation of the hollow colony (Molinari-Novoa et al., 2021). Currently, seven freshwater and marine species are recognized for the genus, with sequence data available on GenBank only for *C*. *adriatica* (marine) and *C*. *regularis* (freshwater) (Broady & Ingerfeld, 1991; Fukuoka et al., 2022). Morphologically and ecologically, our populations are similar to *C*. *regularis*, but they were not recovered as the sister clade of this species, suggesting that they could constitute a new species.

The Meco population is genetically and morphologically related to the genus *Oxynema* (Chatchawan et al., 2012). However, due to the low bootstrap value obtained for this relationship in the phylogeny, and considering the latter’s ecology of halophilic, thermal habitats or warmer regions, further taxonomic analyses are necessary to clarify its taxonomic identity.

The population from El Carrizal was recovered in the phylogeny as closely related to *Ancylothrix rivularis*, proposed by Martins et al., (2016). The morphology also showed similarities to this taxon, with cylindrical, uniseriate trichomes, often slightly constricted at the cross-walls while attenuated and bent at the ends, conical-rounded, narrow apical cells, and an absent calyptra, validating that these populations belong to the genus. Their ecology is similar to *A*. *rivularis* as well. However, *A*. *rivularis* was not recovered as the sister clade to our samples, so additional taxonomic studies are needed for species identification.

The samples of *Oxynema* and *Ancylothrix* presented herein represent the first records of these taxa for Mexico and seem to have a distribution restricted to calcareous basins.

The *Wilmottia* aff. *murrayi* populations are phylogenetically similar to and present the diagnostic characters of the genus, including narrower trichomes with non-attenuated ends, absence of calyptra, and distinct constrictions at cross–walls (Strunecký et al., 2011). This genotype is endemic to Antarctica, but the populations studied here coincide with the reported ecology of the genus in growing on the margins of cold-water rivers.

The Meco population was recovered phylogenetically within the Rivulariaceae family. In morphological and ecological terms, it resembles the description provided by Gardner (1927) as *Dichothrix* aff. *willei*. The relatively low percentage of genetic affinity shared with the published sequences of species in GenBank suggests that it is a new species that requires further taxonomic studies for species validation.

Currently, the main criterion for classifying cyanobacteria is based on its phylogenetic position. However, the data for numerous groups remain incomplete, as many species have not been sequenced or their sequences are not available in the public repositories. Despite the taxonomic uncertainty of some of the populations in this study, we recognize that they may represent potential new species inhabiting mountain rivers in tropical regions, which display an ecology unique to the rest of the world’s cyanobacteria species.

Once the affinities and environmental preferences of the studied populations have been established and the taxonomic identities have been defined, some cases require more studies, denoting the possibility of new records for Mexico and the world. This study emphasizes the importance of benthic species as dominant in the growth of tropical rivers and suggests the need for them to be named with explicit reference to the particularities that allow the recognition of mats with toxigenic potential in lotic environments.

We propose the definition and use of the term Cyanobacteria Harmful Algal Mats (CyanoHAMs) defines growths of cyanobacteria that develop in the benthos and that can generate a large biomass with potentially toxic implications to mammals, including humans (Duana et al., 2022; Legleiter & Hodges, 2022; Rider et al., 2024). In general, growths are easily recognized in lentic environments because they are suspended in the water column, their color changes to a bluish-green tone, and they generally form a thick, viscous layer on the surface (Sanseverino et al*.*, 2016; Frau, 2023). In lotic environments, on the other hand, the frequent and massive cyanobacterial growths can encompass different textures and may extend over large patches of the sediment in different parts of the world (Gaget et al., 2022). Furthermore, they are often barely perceptible to the human eye due to the depth of the channel, the turbulence generated by the water flow, and/or the high concentration of dissolved solids (Bojorge García et al., 2010; Cartajena-Alcántara et al., 2020). As such, the term CyanoHAMs is proposed for benthic populations that grow massively at the bottom of rivers. These growths are not necessarily considered planktonic blooms and may not cover the entire benthic surface of the river, since the type and amount of available substrate may vary as environmental factors (Aguilera et al., 2023; Robichon et al., 2023). Regarding quantity of these mats, authors such as Frau, (2023) and Quiblier et al., (2021) state that one of the most important elements in defining blooms is their quantum measurements. The proposal by Quiblier and collaborators (2021) to benthic growths mentions that for coverage up to 20%, a level of surveillance must be maintained for conditions that may trigger greater cyanobacterial growths. Coverage between 20 and 50% requires maintaining an alert mode by increasing monitoring and informing the local population. Coverage greater than 50% requires a specific mode of action that depends on the unique characteristics of the mats, such as whether or not it is detached from the substrate. The results of the present study show that potentially toxin-producing populations have significant percent coverages from 15 to 40%. As these populations inhabit mountain streams with low flow, we propose lowering the afore mentioned threshold for starting the alert mode from 20 to 15%. Likewise, we propose that the surveillance mode should encompass coverages from 15 to 40%, and coverage greater than 40% constitute the action level. These thresholds take into account the human populations in close proximity to the studied rivers that use the water directly for consumption and that the risk to human health is greater in these cases.

An important concern in determining these alert/action levels are the methods used for the detection and quantification of growths. Methodologies have innate problems that correspond not only to their standardization, but also to the difficulty that developing countries face with respect to inequality in scientific development, gender biases, access to funding, and differences in technical capabilities between countries (Aguilera et al., 2023; Almuhtaram et al., 2021). The use of a more precise definition for toxic cyanobacterial massive growths occurring in lotic environments is an important step for recognizing four aspects in their characterization: (1) detection of cyanobacterial growths with percent coverages greater than 15%; (2) phenotypic and genotypic identification of species involved in the massive growths for the detection of new toxin-producing taxa, (3) standardize chemical methods that validates the presence of some type of cyanotoxin, and (4) associate these species with the production of metabolites and their impact on the deterioration of water quality and, consequently, the greater ecosystem services provided by the basins.

In the present study, the presence of *mcy*E and *ana*F was found in *Cyanoplacoma* sp., while only *ana*F was found in *Wilmottia* aff. *willei* and *Oxynema* sp. As some authors have reported the possibility that the same species may simultaneously produce more than one toxin (Buratti et al., 2017), future research should confirm the capacity of *Cyanoplacoma* to synthesize both toxins. We note that these three species develop in oligotrophic rather than eutrophic sites although with contributions of diffuse nutrients loads that come from nearby human activities. Although these rivers (AB, SD, and ME) are located within subbasins that have conserved forest areas, but are subject to physical alterations of the channel for local water extraction, for example. These changes in water flow due to diversions or damming represent an important component in the proliferation of cyanobacteria and can be added synergistically to other factors such as small nutrient inputs from human activities (Robichon et al., 2023; Schulte et al., 2022). On the other hand, *Cyanoplacoma* sp. at the SM site was recorded minimally disturbed, showing, as in other studies, that different combinations of land uses can host toxin-producing cyanobacteria (Rider et al., 2024; Schulte et al., 2022). These results may also be related to strategies of competition for substrate with other bacteria, algae, or even as a measure to avoid herbivory (Bouma-Gregson et al., 2018; Graham et al., 2020; Kim et al., 2021; Reinl et al., 2021; Timoshkin et al., 2016). The production of toxins, such as *ana*F expression, may also be influenced by the N and P concentrations. The cellular production of anatoxin has been suggested to be stimulated by moderate N deficiency (Colas et al., 2021; Massey et al., 2022;). Likewise, this production may depend on the form of available N, which is slightly higher when NH_4_–N predominates, compared to a medium enriched with N-NO_3_ or urea-nitrogen (Yadav et al., 2022). Despite this, our results found that water deficient in nutrients (Table 1, DIN ratio: SRP = 1.25 and 1.70 mg/L) had the highest concentration of N in the form of nitrates (1.25 and 1.70 mg/L). This is likely related to metabolic strategies for incorporating P and N that differ from the strategies used by planktonic cyanobacteria (Chorus et al., 2021). Another environmental factor that can alter toxin production in cyanobacteria is water temperature (Colas et al., 2021). In lentic environments, the highest intracellular anatoxin concentrations have been recorded at colder temperatures between 19 and 21 °C. When temperatures rise too high, anatoxin production decreases, with a minimum observed at 30 °C (Colas et al., 2021). The water temperatures recorded in this study where cyanobacteria with potential production of cyanotoxins were recorded ranged very widely from 7.6 to 25.6 °C. Other biological factors such as bacteriological competition and/or predation also likely play an important role in toxin production (Bouma-Gregson et al., 2019: Lamka et al., 2023). The *Cyanoplacoma* and *Wilmottia* mats presented the lowest percent coverages, which indicates that massive growths are not necessarily required for toxin synthesis and consequently risks to ecological and human health. Mats dominated by Chroococcales and Oscillatoriales have been shown to possess toxin-producing capacities (Quiblier et al., 2013) and have been reported as toxic in rivers across Europe (Aboal et al., 2005). In this study, Nostocales did not show toxin-producing genes, but they have been reported for some *Nostoc*-producing species (Kurmayer, 2011; Mohamed et al., 2006; Oudra et al., 2009; Smith et al., 2011) that may be very abundant in other lotic environments in the central region of Mexico. As such, we suggest the continued study and characterization of these taxa in the region. Molecular methods can serve as tools for their early detection and characterization, but are not definitive in identifying toxin-producing populations. The new genera recorded with cyanotoxin genes in this study (*Cyanoplacoma* and *Oxynema*) increase the potential risk to both ecosystem and human health, as they inhabit rivers that are closely tied to the livelihood of rural populations (Caro-Borrero et al., 2020), who use the water for domestic and economic tasks such as agriculture and animal husbandry. Therefore, CyanoHAMs can become a risk factor that decrease water quality (Massey et al., 2022; Moreira et al., 2022). Following the arguments presented in this manuscript, we invite other studies to use the concept of cyanoHAMs for classifying massive and potentially hazardous growths in lotic water bodies, and to complement this visual description with analyses that inform not only the taxonomic identifies of the population(s) present within the cyanoHAMs, but also whether or not they are capable of producing toxins.

## Conclusion

The cyanobacteria studied herein can be classified into three groups based on their environmental affinity: (1) cyanobacteria inhabiting waters with a higher concentration of ions (specific conductance 141–1528 µS/cm), such as *Dichothrix* aff*. willei, Nostoc montejanii*, and *Compactonostoc* sp.; (2) cyanobacteria found in cold-temperate waters, such as *Nostoc tlalocii*, *Wilmottia* aff. *murrayi*, and *Cyanoplacoma* sp.; and (3) cyanobacteria that prefer waters with greater nutrient enrichment (SRP and NID), such as *Ancylothrix* sp. and *Oxynema* sp. Our results show that the second grouping in particular contains taxa with potentially toxin-producing capabilities, allowing us to conclude that eutrophic environments are not necessarily related to such toxin-producing abilities. Temperature and geological origin were also associated with specific adaptations for colonizing the environment, but were not conclusive in differentiating potentially toxin-producing populations. A recurring environmental characteristic of all sampled rivers was high levels of oxygenation in the water. However, small physical alterations of the habitat resulting from urban settlements near rivers were reported in most cases, demonstrating that anthropogenic intervention is a multifactorial element to consider in this type of studies.

The use of phylogenies as a tool for determining the taxonomic identities of cyanobacteria facilitates the recognition of biological diversity that is otherwise often underestimated using morphological descriptions due to similar morphotypes that result from adaptive processes in similar environments. Within the study of toxin-producing benthic cyanobacteria, phylogenetic analyses are essential for recognizing toxic taxa, not only for documenting the diversity of their genotypes but also for distinguishing evolutionary relationships that help us understand the production of toxins in different environmental conditions. Our results show that Mexican mountain rivers harbor significant levels of cryptic cyanobacterial diversity that remain to be characterized, an important task given the intrinsic value of aquatic biodiversity and the potential production of cyanotoxins in local water supplies upon which rural populations directly depend (Caro-Borrero et al., 2020). This study contributes to the recognition of new genera recorded with cyanotoxin genes: *Cyanoplacoma* sp*.* and *Oxynema* sp, which should be included in the global references of potentially producing benthic cynobacteria and which can increase the potential risk to both ecosystem and human health.

The term “Cyanobacteria Harmful Algal Mats” (CyanoHAMs) is herein proposed to refer to the massive growths of cyanobacteria in the benthic environments of rivers. This represents a first step for recognizing the ecological, functional, and structural differences in massive growths from lotic environments compared to lentic ones. The present study only considered the most-commonly reported cyanotoxins in the literature. We cannot exclude the possibility that the studied populations are potential producers or even active producers of other types of toxins, especially when considering that the phylogenetic analyses suggest they may represent new genera and species. These results demonstrate that continued work is needed to identify toxin-producing populations specifically in lotic environments, as those from lentic environments have traditionally been more studied (Quiblier et al., 2013). Likewise, we stress the importance of using a combination of techniques for detecting, quantifying, and identifying these toxins. These include the use of molecular methods that can recognize potential risky taxa, physical–chemical methods that can identify and quantify the different cyanotoxins and their congeners, and finally bioassays that allow us to perceive these effects on the greater aquatic communities. These strategies allow for a detailed evaluation of the role of invertebrate organisms within the riparian trophic chain, as well as the potential risks to human health.

### Supplementary Information

Below is the link to the electronic supplementary material.

Supplementary file1 (DOCX 16 KB)

## Data Availability

Not applicable
