# Supplementary material for: Cyanobacterial Harmful Algal Mats (CyanoHAMs) in tropical rivers of central Mexico and their potential risks through toxin production
Source: Environ Monit Assess. 2024 Apr 2;196(4):408. doi: 10.1007/s10661-024-12568-4 (PMC10984904; doi:10.1007/s10661-024-12568-4)
Supplement: Supplementary file 1 — Supplementary file1 (DOCX 16 KB) [file 10661_2024_12568_MOESM1_ESM.docx]

Supplementary material. Taxonomic assignment to study populations.

| Taxonomic assignment | Description |
| --- | --- |
| *Cyanoplacoma* sp. (Broady et Ingerfeld 1991) | Compact colonies spherical or cerebriform, brown, 1 to 30 mm high. Cells spherical or polygonal, arranged in cubical patterns in 4 to 5 layers in the surface or irregular interiorly. Cells 3.3 to 3.8 μm in diameter at surface and intermediate layers, 4.3 to 5.7 μm in diameter in the inner layer. |
| *Wilmottia* aff*. murrayi* (W. et G.S.West) (Strunecký, Komárek et Elster 2011) | Filaments arranged in fine mats, blue–green and 10 to 15 cm in length. Filaments solitary or fascicles parallelly arranged. Very fine and diffluent slimy, colorless, envelope. Trichomes straight or slightly curved, sometimes slightly constricted at cross walls. Cells 3.0-6.0 μm wide, 2.5-6.5μm long, pale blue green, with homogeneous, finely granular content, usually with scattered large granules which are sometimes constricted near cross walls. |
| *Ancylothrix* sp. (Martins et Branco 2016) | Filaments forming dark green mats. Sheaths rare, thin and colorless, 4.5–7.0 μm wide. Trichomes cylindrical, sometimes slightly constricted at the cross-walls, attenuated and bent at the ends, 5.0–6.0 μm wide. Cells shorter than wide to isodiametric, 2.0–5.0μm long. Apical cells conical and narrowed. |
| *Oxynema* sp. (Chatchawan, Komárek, Strunecký, Šmarda et Peerapornpisal 2012) | Filaments forming bright blue green mats. Sheaths rare, fine, firm and colorless. Trichomes cylindrical, not constricted or later, 3.5-8 μm wide, shortly attenuated at the ends. With terminal cells more elongated, conically narrowed and sharply pointed, always without calyptra. |
| *Nostoc* *montejanii* (Carmona, Caro et Becerra 2023) | Colonies spherical, dark to gray green. Within the colony, filaments segmented into several small groups that show compartmentalization of mucilage, later forming a small and compact spherical colony. Released filaments covered with a compact and stratified sheath, with filaments or multiseriate filaments later becoming densely entangled to form young colony. Sheath thick, colorless. Vegetative cells short, barrel-shaped to subspherical, 5 μm long and 6.1 μm wide. Heterocytes spherical, 5.2 μm in diameter. |
| *Nostoc* *tlalocii* (Carmona, Caro et Becerra 2023) | Colonies spherical or transformed to ear-shaped via larva stage association with chironomids. Gray-green color colonies throughout development. Within colonies, filaments segmented into several small groups, which show compartmentalization of mucilage and later form small spherical colonies. Filaments released by rupture of colony, covered with sheaths, and become densely entangled to form young colony. Vegetative cells subspherical or oblong, 5.0 μm long and 4.2 μm wide. Heterocytes spherical, 3.5-4.6 μm in diameter. |
| *Compactonostoc* sp. | Colonies sub-spherical in juvenile to globose in adult stage, brown. Within colonies, filaments segmented into several small groups of uniseriate or multiseriate cells, the small groups showing compartmentalization of mucilage and later forming small spherical colonies. Filaments released by rupture of colony. Sheath thick, colorless. Vegetative cells short, barrel-shaped to subspherical, 4 μm long and 4 μm wide. Heterocytes spherical, 3-4.5 μm in diameter. |
| *Dichothrix* aff *willei*  (N.L.Gardner 1927) | Colonies widened in the form of fasciculate clusters. Filaments up to 2 mm long, 7-10 μm wide. Sheaths thin, narrow, and striated, colorless to yellow. Trichomes cylindrical, narrowed towards ends, not forming the typical hairs. Heterocytes hemispherical, of the same diameter as the trichome. |
